# On the Primary Ionization Mechanism(s) in Matrix-Assisted Laser Desorption Ionization

**DOI:** 10.1155/2012/161865

**Published:** 2012-11-27

**Authors:** Laura Molin, Roberta Seraglia, Zbigniew Czarnocki, Jan K. Maurin, Franciszek A. Pluciński, Pietro Traldi

**Affiliations:** ^1^National Council of Researches, Institute of Molecular Sciences and Technologies, Corso Stati Uniti 4, I35100 Padova, Italy; ^2^Faculty of Chemistry, University of Warsaw, Pasteura 1, 02-093 Warsaw, Poland; ^3^National Medicines Institute, Chełmska 30/34, 00-725 Warsaw, Poland; ^4^National Centre for Nuclear Research, 05-400 Otwock, Świerk, Poland

## Abstract

A mechanism is proposed for the first step of ionization occurring in matrix-assisted laser desorption ionization, leading to protonated and deprotonated matrix (Ma) molecules ([Ma + H]^+^ and [Ma − H]^−^ ions). It is based on observation that in solid state, for carboxyl-containing MALDI matrices, the molecules form strong hydrogen bonds and their carboxylic groups can act as both donors and acceptors. This behavior leads to stable dimeric structures. The laser irradiation leads to the cleavage of these hydrogen bonds, and theoretical calculations show that both [Ma + H]^+^ and [Ma − H]^−^ ions can be formed through a two-photon absorption process. Alternatively, by the absorption of one photon only, a heterodissociation of one of the O–H bonds can lead to a stable structure containing both cationic and anionic sites. This structure could be considered an intermediate that, through the absorption of a further photon, leads to the formation of matrix ions. Some experiments have been performed to evaluate the role of thermal ionization and indicate that its effect is negligible. Some differences have been observed for different matrices in the formation of analyte molecule (M) ion [M + H]^+^, [M − H]^−^, M^+•^, and [M − 2H]^-•^, and they have been explained in terms of ionization energies, pKa values, and thermodynamic stability.

## 1. Introduction

Matrix-assisted laser desorption ionization (MALDI) [1] is a method of wide analytical interest, representing an effective approach to obtain information on the molecular weight of macromolecules. Due to the privileged formation of singly charged molecular species ([M + H]^+^, [M − H]^−^, alkali cationized molecules) it leads to information on macromolecules mixtures, even if ion suppression effects can be present to some extent. MALDI is widely employed in the protein field and is successfully used to determine the intact protein molecular weight up to 10^5^ Da, as well as to obtain information on the protein sequences by a rapid analysis of their digestion products and by MS/MS experiments performed on them.

Different mechanisms have been proposed to explain MALDI ionization [2]. All are in agreement with the presence of two different steps: (i) primary ion formation from the matrix (Ma); (ii) secondary ion formation of the analyte (A) originating from the gas phase interaction of reactive matrix ions and neutral molecules of the analyte. Point (i) is the most debated one, due to the fact that the photon energy (for N_2_ laser *hν* = 3.68 eV, for triplicated Nd:Yag laser *hν* = 3.55 eV) is much lower than that required for matrix ionization (typical ionization energies (IEs) of the most employed matrices are in the range 7–10 eV) [3].

It has been proposed [4] that multiple photon absorption, leading to multiple electronic excited matrix molecules, followed by energy pooling phenomena, would lead to the formation of the matrix odd electron molecular ion Ma^+•^ that, in turn, reacts with neutral matrix molecule leading to [Ma + H]^+^. The last species must be considered responsible for the analyte (A) protonation in gas phase:

(1) $$\begin{array}{c} {\text{MaH}}^{+}+\text{A}\to \text{Ma}+{\text{AH}}^{+} \end{array}$$

only if PA_A_ > PA_Ma_, (PA = Proton Affinity).

The possible limitation of this mechanism lies in the low probability of multiphoton absorption and energy pooling, which makes the high ionic yield observed in MALDI experiments difficult to explain. 

Alternatively it has been considered that the electronic excitation (1 photon required) leads in general to an increase of the matrix acidity, promoting proton transfer. However, excited-state proton transfer (ESPT) [1, 5] does not affect carboxylic acids, and consequently it cannot be invoked as effective mechanism for proton transfer for the MALDI matrices; furthermore ESPT is active for compounds that do not exhibit any activity as MALDI matrix.

The desorption of preformed ions, already present in the solid state sample (before laser irradiation), has been proposed as MALDI ionization mechanism [6]. The laser effect would be, in this case, just the desorption of these ions or matrix clusters containing them. In the latter case ions are made free by declustering reactions occurring in the high density desorbed plume. This mechanism can be invoked in the case of preprotonated compounds, metal complexes, and ionic compounds, and it is well supported by thermal desorption experiments, revealing the presence in gas phase of the same ions generated by laser irradiation. However the presence of MaH^+^ ions in the matrix before laser irradiation is difficult to prove, and consequently a gas phase protonation mechanism different to that reported in (1) must be invoked.

Other ionization mechanisms have been proposed as laser-induced shockwaves [7] (the laser irradiation produces local high pressure shocks, resulting in the thermal excitation of a microvolume of the matrix-analyte sample), thermal ionization [8] (originating by the photon-phonon transformation in the crystal lattice), and ion-electron emission due to piezoelectric effects [9]. 

All the above described proposals are mainly focused on the physical aspects of MALDI ionization mechanism and the chemical nature of the matrix has been only marginally considered [10].

In this paper we propose and discuss another possible mechanism, related to the chemical nature of the matrices effective for MALDI experiments and their structural arrangements when crystallized. The solid-state interaction of carboxylic groups present in the matrix molecules could be considered responsible, when irradiated, for some disproportionation reactions, leading to both [Ma + H]^+^ and [Ma − H]^−^ ions, that is, the reactants necessary for the [A + H]^+^ and/or [A − H]^−^ formation. Little is known on this aspect but measurements on gas-phase basicity of some matrix neutrals and anions [11, 12] indicate that disproportion reaction can be available through a two-photon absorption. This hypothesis has been investigated both from the experimental and from a theoretical point of view. 

## 2. Experimental

### 2.1. Sample Preparation

One *μ*L of three different matrix solutions (*α*-cyano-4-hydroxycinnamic acid (HCCA), 2,5-dihydroxybenzoic acid (DHB), and sinapinic acid (SA) (10 mg/mL acetonitrile)) was deposited on the MALDI plate and left to dry. 

The three matrix solutions were mixed with a Cobalt powder suspension (10 mg/mL in H_2_O) in order to obtain “matrix : Cobalt” molar ratio: 10 : 1, 1 : 1, and 1 : 10 (w/w). One *μ*L of the three solutions so obtained was deposited on the MALDI plate and left to dry. 

0.01 mg of insulin were dissolved in 1 mL of four different solutions (H_2_O, H_2_O containing 0.1% formic acid, H_2_O containing 0.1% acetic acid, and H_2_O containing 0.1% trifluoracetic acid). 5 *μ*L of each insulin solution obtained were mixed with 5 *μ*L of a sinapinic solution (10 mg/mL in H_2_O/ACN, 1 : 1, v/v). One *μ*L of the solutions so obtained was deposited on the MALDI plate and left to dry. 

0.1 mg of human serum albumin (HSA) were dissolved in 1 mL of four different solutions (H_2_O, H_2_O containing 0.1% formic acid, H_2_O containing 0.1% acetic acid, and H_2_O containing 0.1% trifluoracetic acid). 5 *μ*L of each HSA solution obtained were mixed with 5 *μ*L of a sinapinic solution (10 mg/mL in H_2_O/ACN, 1 : 1, v/v). One *μ*L of the solutions so obtained was deposited on the MALDI plate and left to dry. 

### 2.2. MALDI-MS Measurements

MALDI/MS measurements were performed using a MALDI-TOF UltrafleXtreme (Bruker Daltonics, Bremen, Germany), equipped with 1 kHz smart beam II laser (*λ* = 355 nm) and operating in the positive and negative reflectron ion modes. 

The instrumental conditions employed to analyze molecular species in the *m/z* range 50–3000 in positive ion mode were Ion Source 1: 25.00 kV; Ion Source 2: 22.30 kV, Lens: 7.70 kV, Pulsed ion extraction: 80 ns, Reflector: 26.45 kV, Reflector 2: 13.45 kV. The instrumental conditions employed to analyze molecular weight in the *m/z* range 980–7000 were Ion Source 1: 25.00 kV; Ion Source 2: 22.40 kV, Lens: 8.00 kV, Reflector: 26.45 kV, Reflector 2: 13.45 kV, Pulsed ion extraction: 120 ns. The instrumental conditions employed to analyze molecular weight in the *m/z* range 50000–80000 were Ion Source 1: 25.00 kV; Ion Source 2: 23.30 kV, Lens: 6.50 kV, Pulsed ion extraction: 450 ns.

The instrumental conditions to analyze low molecular weight in the *m/z* range 50–2000 in negative ion mode were Ion Source 1: 20.00 kV; Ion Source 2: 17.85 kV, Lens: 6.15 kV, Reflector: 21.15 kV, Reflector 2: 10.75 kV, Pulsed ion extraction: 80 ns.

External mass calibration was done using the Peptide Calibration Standard, based on the monoisotopic values of [M + H]^+^ of Angiotensin II, Angiotensin I, Substance P, Bombesin, ACTH clip (1–17), ACTH clip (18–39), Somatostatin 28 at *m/z* 1046.5420, 1296.6853, 1347.7361, 1619.8230, 2093.0868, 2465.1990, and 3147.4714, respectively, and by using the monoisotopic values of [M + H]^+^ of insulin at “mass/charge” (*m/z*) 5730.6081.

The [Ma + H]^+^ and [Ma − H]^−^ ion production has been tested by irradiating the matrix sample with the same laser power and with the same number of laser shots (2000).

### 2.3. Theoretical Calculations

The calculation of energy of the photo processes postulated below was performed according to the thermodynamic Hess' law. The formation energy of the products in particular photoreactions was calculated with simultaneous optimization of its geometry. The dimer structures taken from the Cambridge Structural Database were treated as optimal. All values of the formation energy were calculated by DFT method applying the hybrid functional B3LYP and 6-31+G* function base [13]. This function base contains diffuse functions (designed by “+”) and polarization ones (designed by “∗”). These supplementary functions make the essential 6-31G base capable to describe correctly the ionic systems. The calculations were done using Spartan software [14].

## 3. Results and Discussion

It is interesting to note that the most of effective matrices (Ma) to perform MALDI experiments contain the carboxyl group. At first sight this aspect could suggest that acidity of the matrix directly affects the production of protonated molecules [A + H]^+^ of the analyte. However it is emphasized that this behaviour cannot explain how, with the same acidic matrix, deprotonation reactions can take place, with the formation of [A − H]^−^ ions of the analyte. As discussed in the introduction, the species responsible for the generation of [A + H]^+^ is considered, in the most of the cases, to be [Ma + H]^+^, that is, the protonated matrix, and not the Ma molecules and/or Ma^+•^. Consequently the [Ma + H]^+^ production should be considered to be the focal point of the MALDI mechanism. As above discussed, the most of MALDI mechanisms proposed in the literature are mainly focused on physical aspects related to laser irradiation [2]: in the case of pooling mechanism [4] the interaction of the solid sample with the photons leads to an electronic excitation that, only after pooling phenomena, can give account for Ma^+•^ (and not [Ma + H]^+^) ion production. In the case of preformed ion mechanism, the ions are considered to be already present in the solid-state sample, and the only effect of laser irradiation is to make them free in gas phase [6].

**Figure 1:**
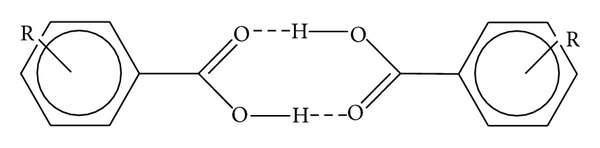
Dimeric structure of carboxylic acids with the formation of centrosymmetric 8-membered ring due to the presence of hydrogen bonds.

Here we propose a different mechanism, in which, considering the molecular arrangement in solid state of carboxylic acids, a possible interaction between the carboxylic groups is present, as shown in Figure 1. Carboxylic acids are known as compounds which very readily form strong hydrogen bonds, and their carboxylic groups can act as both donors and acceptors [15–17]. In some cases intermolecular hydrogen bonds between such groups are formed. In the Cambridge Structural Database version 5.32 (Conquest version 1.13) [18] as much as 4129 (out of 17615 incorporating carboxyl groups) structures containing intermolecular carboxyl-carboxyl hydrogen bonds can be found. Most of them (3431 structures) form centrosymmetric or quasicentrosymmetric 8-membered ring arrangements described by the graph descriptor *R*_2_^2^(8) [19–21]. The others form chain structures, including catemer arrangements where the H-bonds are formed between translation symmetry-related molecules (including screw axes symmetry). This feature is characteristic only for chiral compounds which must crystallize in the chiral space groups where the symmetry centre is absent, but also in the case of some simple carboxylic acids, for example, formic [22, 23] and acetic [22, 24] acids. The preference to form dimeric versus catemer structures is not clear while comparing the structures of acetic [22], trifluoro- [25], and trichloro-acetic acid [26]. 

**Figure 2:**
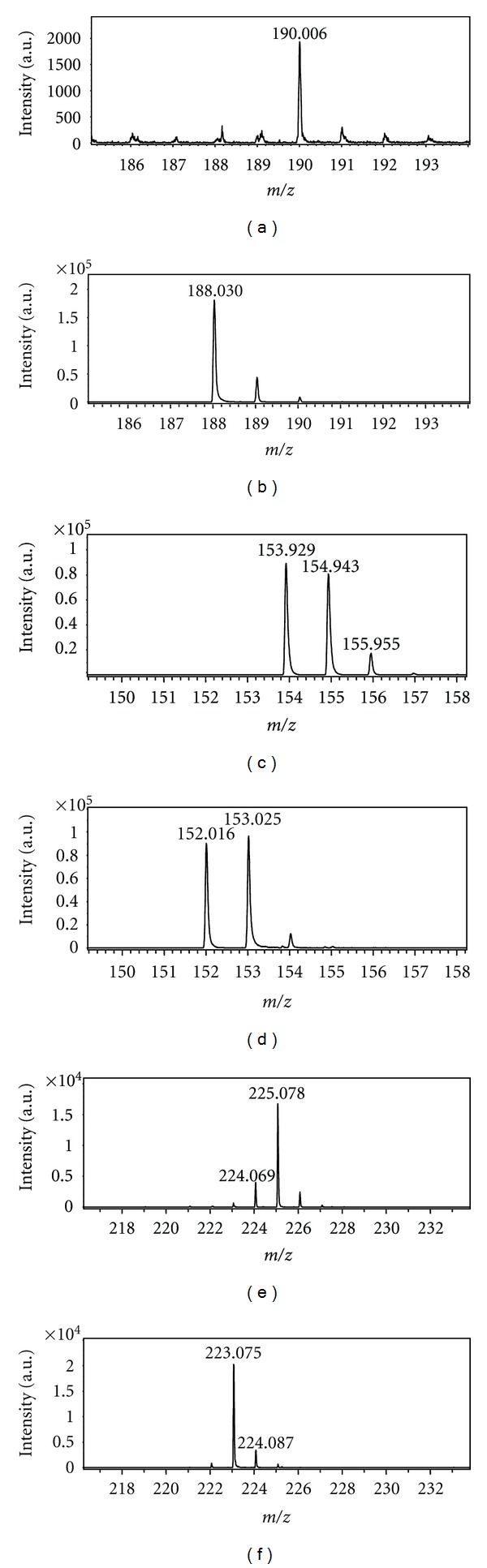
Molecular ionic species (positive ions: (a), (c), (e); negative ions: (b), (d), (f)) generated by laser irradiation of HCCA, DHB, and SA.

**Scheme 1:**
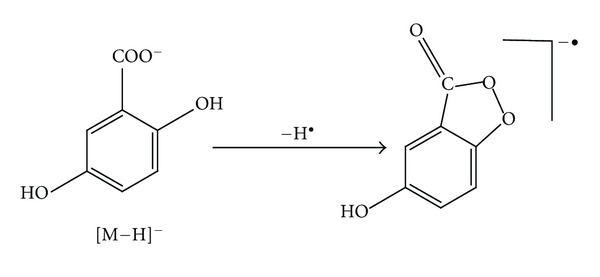
Formation of [M − 2H]^−•^ from [M − H]^−^ anion by H^•^ loss.

The laser irradiation of the matrix dimer with the structure shown in Figure 1 could be considered responsible for the formation of the ion couple [Ma + H]^+^ and [Ma − H]^−^. Indeed, by irradiation of sinapinic acid, both [Ma + H]^+^ and [Ma − H]^−^ ions are produced with practically the same abundances. The related spectra, reported in Figures 2(e) and 2(f), have been obtained by using the same laser power (35%) and the same delay time (80 ns). The situation is more complicated in the case of DHB (Figures 2(c) and 2(d)): [Ma + H]^+^ and [Ma − H]^−^ ions with similar abundance (80700; 96500, resp.) are produced, suggesting the occurrence of the disproportionation reaction, but also Ma^+•^ and [Ma − 2H]^−•^ or [M − H_2_]^−•^ ions are present with practically identical abundance. The formation of the former can be justified by the low ionization energy of 2,5-DHB (8.05 eV) lower than that of SA (8.47 eV) and HCCA (8.50 eV) [10], while the [Ma − 2H]^−•^ formation can be due to the high stability of the radical anion produced through H^•^ loss from [Ma − H]^−^ anion. (see Scheme 1). In the case of HCCA the formation of [Ma − H]^−^ ion is highly favoured (ion abundances 200000), while [Ma + H]^+^ ion exhibits an abundance of 2000. This can be justified by its pKa value (around 8), higher than that of other matrices (around 4) [27].

The main question that arises is as follows: is this dissociation reaction activated by photons or by the local heating due to laser irradiation? Thermal ionization could occur, as described by Dreisewerd et al. [8] in the matrix bulk, following the rules of surface ionization. The typical reaction observed by thermal ionization is a disproportion, governed by the Saha-Langmuir equation (2) in which the work function is replaced by the matrix electron affinity (EA):

(2) $$\begin{array}{c} 2\text{Ma}\overset{\Delta H}{\to }{\text{Ma}}^{-}+{\text{Ma}}^{+},\\ \alpha_{i}={\text{Ce}}^{\left(\text{EA}-\text{IP}\right)/kT}, \end{array}$$

where *α*_*i*_ is the extent of ionization of species *I*, C is a constant near unity, EA is the matrix electron affinity, IP is the matrix ionization potential, *k* is the Boltzmann constant, and *T* is the absolute temperature. 

**Figure 3:**
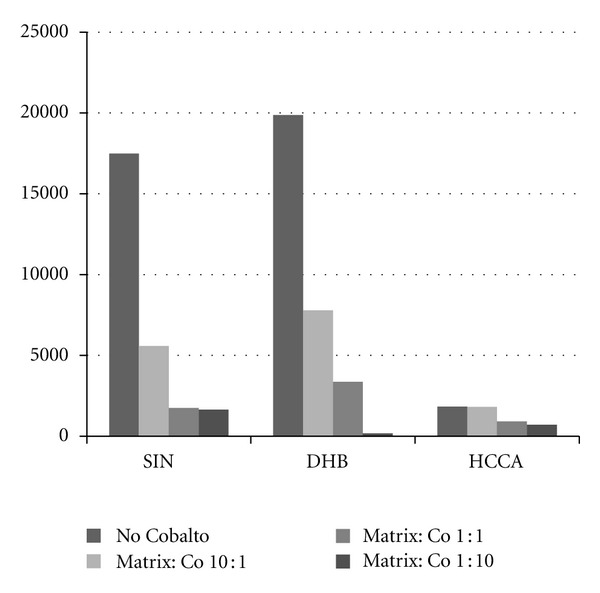
[Ma + H]^+^ abundance of the different matrices mixed with increasing amounts of powdered cobalt.

From this equation it follows that thermal ionization in MALDI would require very high temperature, not available by simple irradiation of the matrix. To investigate this point the three matrices under investigation were mixed with powdered cobalt so as to increase the laser power absorption. In these conditions the role of thermal effects would be further enhanced. The results so obtained are reported in the histogram of Figure 3. It can be seen that the presence of cobalt leads to a decrease of [Ma + H]^+^ ion formation, and so the possible increase of temperature related to the higher energy absorption in the presence of cobalt cannot be responsible for the [Ma + H]^+^ ion production.

In order to evaluate the role of photon irradiation, some theoretical calculations were performed. Three calculation models of the primary matrix ionization mechanisms for UV-MALDI were proposed that might generate analyte ions. In these model calculations benzoic acid dimer and benzoic acid derivatives dimers 2,5-dihydroxybenzoic acid, 4-methoxybenzoic acid, 4-nitrobenzoic acid, and 4-N,N-dimethylaminobenzoic acid were tested in terms of their properties as potential matrix compounds. Benzoic acid derivatives were proposed to allow comparison of the electron-donor or electron-acceptor properties of the substituents on the ability to ionize the analyte molecule. The structures of dimer molecules were taken from Cambridge Structural Data Base and used as the starting crystal structures in the quantum chemical calculations of their ionization energies. 

**Scheme 2:**
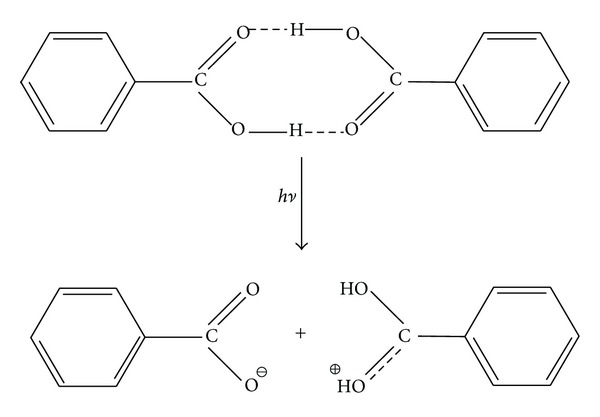
First ionization model.

According to the first model, an absorbed UV photon by the matrix dimer induces its dissociation process into monomers accompanied by its simultaneous heterolysis of one of the hydrogen bonds. This calculation model in the case of benzoic acid (as an example) is illustrated by the dissociation reaction reported in Scheme 2.

 The energies (per one dimer molecule) of all studied reactions *E* were calculated by DFT method at B3LYP (6-+31G*) level and compared with the energies of the photons of two typically used UV lasers emitting pulses of 337 and 355 nm wavelengths *E*_337_ and *E*_355_. These photon energies are *E*_337_ = 3.68 eV and *E*_355_ = 3.50 eV. 

The values of energy calculated for the studied dimers are given in Table 1. These results unambiguously show that this type of dissociation cannot be a one-photon process, and it meets the energetic criterion for the two-photon process, making the probability of such process low. However it is interesting to note that the two-photon process is that invoked by Zenobi and Knochenmuss [10] as the first step of their theory of ionization by pooling phenomena. The data reported in Table 1 indicate that the interaction with two photons is enough to activate the ion pair formation shown in Scheme 2. The experimental data reported in Figure 2 and showing a practically identical abundance of [Ma + H]^+^ and [Ma − H]^−^ for sinapinic acid and for DHB support this mechanism.

**Scheme 3:**
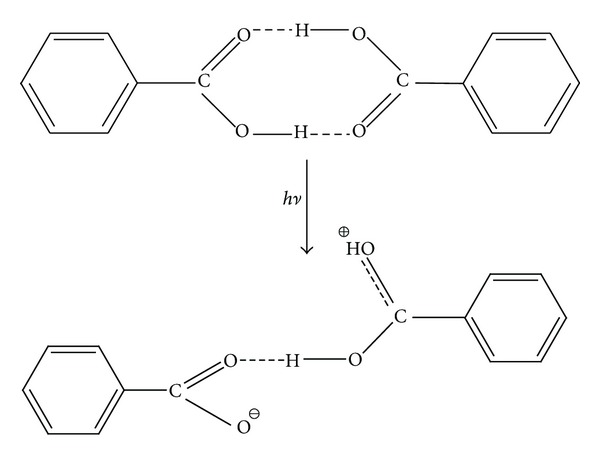
Second ionization model.

The second proposed photoionization mechanism of the matrix dimer molecules corresponds to the reaction reported in Scheme 3. The energies of two processes, a heterodissociation of one of the both O–H bonds associated with the proton transfer to carbonyl oxygen atom and a rotation of the benzoic acid moiety about the O–H hydrogen bond of 180°, contribute to the energy requirement of this reaction. The assumption of such rotation in modelling of this ion was necessary since the nonrotated ion would be unstable enough tending to return to the starting dimer structure during the optimization. The calculated values of the energies are given in Table 2. This process could be invoked for HCCA, due to its high pKa value.

**Scheme 4:**
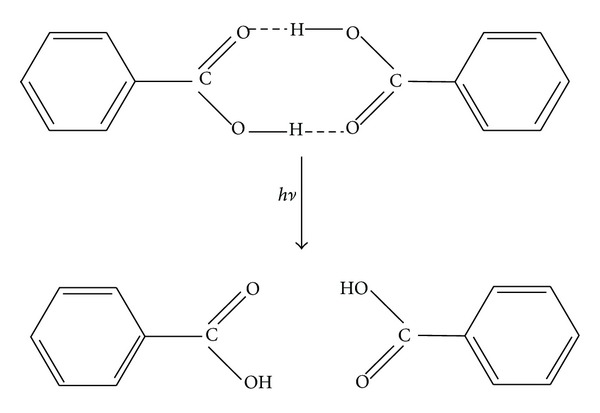
Dissociation of the dimer.

The third photoprocess studied was the homolytic dissociation of the dimers into neutral monomers (Scheme 4). Obviously, this is not ionization process itself but it is reasonable to assume that the proton can be transferred through an excited-state proton transfer [5] from the monomer to the analyte molecule and, due to this, may cause the ionization of the analyte. It is also possible, although less probable, that this is two-stage process. According to this supposition, the first absorbed photon induces the reaction of dissociation of the dimer into monomers (above reaction) and on the second stage, the next photon could cause the ionization of the monomer. The values of the energies for the dissociation of the studied dimers are collected in Table 3.

 The comparison of the photon energy (*E*_355_ = 3.50 eV) with the particular energy of each process studied leads to the conclusion that the one-photon ionization of the matrix dimers can take place according to the second ionization model (Scheme 3) and could operate only for 2,5-dihydroxybenzoic acid, 4-methoxybenzoic acid, 4-nitrobenzoic acid, and 4-N,N-dimethylaminobenzoic acid. It seems that the two-stage ionization of an analyte is also probable to some extent since the life-time of the monomers may be long enough that these monomers could be ionized by the next photon absorbed. 

The analysis of the substituent effect on the calculated energy values (*E*) for the studied compounds leads to the conclusion that there is no correlation between selected properties of the substituents (e.g., electron affinity) and the ability of these compounds to undergo ionization. Of course, by having more data it would be possible to search for such a trend. 

**Figure 4:**
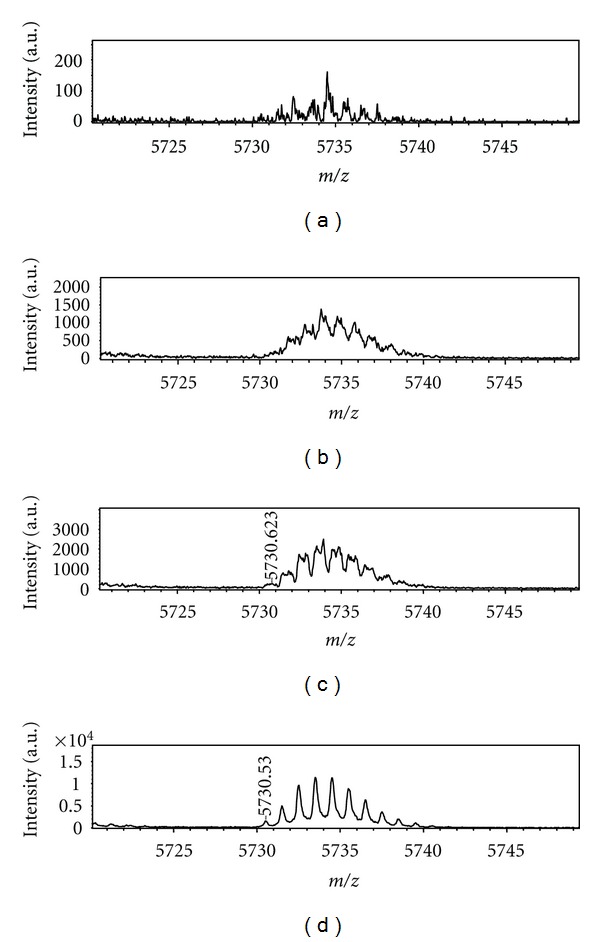
MALDI mass spectra of Insulin obtained by (a) sinapinic acid only, (b) sinapinic acid with 0.1% of formic acid, (c) sinapinic acid with 0.1% of acetic acid, and (d) sinapinic acid with 0.1% of trifluoroacetic acid.

**Figure 5:**
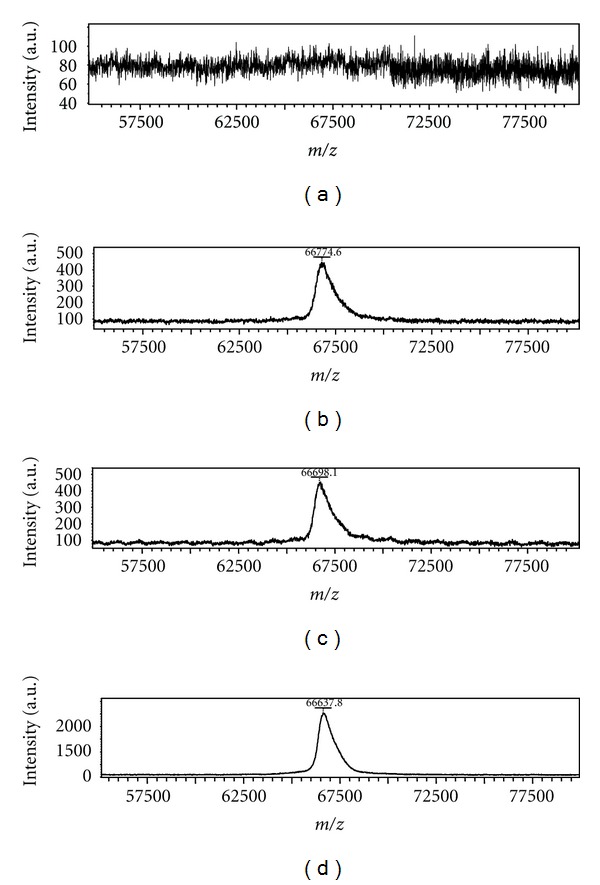
MALDI mass spectra of Human Serum Albumin obtained by (a) sinapinic acid only, (b) sinapinic acid with 0.1% of formic acid, (c) sinapinic acid with 0.1% of acetic acid, and (d) sinapinic acid with 0.1% of trifluoroacetic acid.

**Scheme 5:**
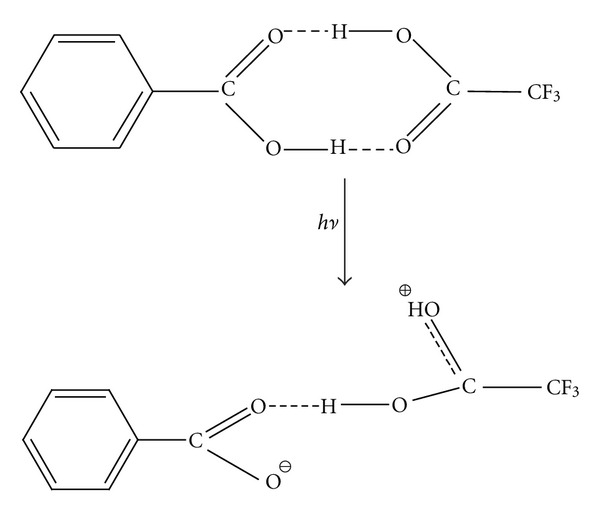
Ionization model of a conjugate of a benzoic acid with trifluoroacetic acid (TFA).

**Figure 6:**
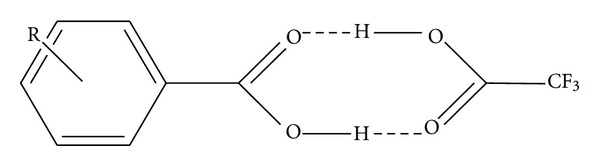
Bimolecular structure due to interaction between substituted aromatic carboxylic acid and trifluoracetic acid.

Finally we investigate the role of small organic acids (Ac) usually added to the (Ma + A) solution before the MALDI measurements. Usually trifluoroacetic acid (TFA) is added at 0.1%, leading to a high enhancement of the analyte ion intensity. To verify this aspect solutions of three matrices (HCCA, DHB, and SA) containing Insulin or Albumin were added with 0.1% of Formic acid (HCOOH), Acetic acid (CH_3_COOH), and Trifluoracetic acid (TFA). The results are shown in Figures 4 and 5, respectively, indicating that with TFA a wide increase of signal intensity is obtained. This could be due to a protonation reaction between TFA and the analyte molecules but, alternatively, it could be considered that the different acidity of TFA could lead to a [Ma + TFA] complex with different electronic distribution: in this case the formation of [Ma + H]^+^ ions would be strongly favoured (Figure 6), due to the higher acidity of TFA. Some calculations were performed also in this case. All calculations concerning the above conjugate have been performed according to schemes shown in Scheme 2 through Scheme 4. It should be taken into account that in the case of the most probable process shown in Scheme 3, both hydrogen bonds are not equivalent, and therefore the weaker one will be broken (see Scheme 5). The corresponding energies calculated for such process are shown in Table 4.

Also the energy of dissociation of conjugates into substituted benzoic acid cation and TFA anion (Table 5) and into separate acid monomers (Table 6) was calculated. The values collected in Table 5, similarly to those in Table 1, suggest that such processes are not possible as one-photon processes and the dissociation into monomers are low-energy processes.

## 4. Conclusions

The obtained data show that considering the matrix molecules in the solid-state sample in a dimeric structure containing intermolecular carboxyl-carboxyl hydrogen bond in centrosymmetric or quasicentrosymmetric 8-membered ring arrangement, the formation of both [Ma + H]^+^ and [Ma − H]^−^ ions can be justified by the occurrence of a two-photon-induced disproportion reaction. Alternatively one-photon induced process can lead to the opening of the eight-membered ring with the subsequent formation of species containing both cationic and anionic sites. Experimental data showed that the processes leading to [Ma + H]^+^ must be considered to be activated by photons, and thermal ionization seems to inhibit this behaviour. 

It is interesting to observe that the obtained results are somehow well related to the mechanisms already proposed with respect to considering the desorption of clusters containing preformed ions [6]; our data seem to suggest that the ions are not originally present in the solid-state sample, but they are originated by the laser irradiation. In the case of multiple photon absorption followed by pooling phenomena [4], necessary for the Ma^+•^ formation, the data discussed above indicate that it is unnecessary to invoke the energy pooling: in fact the absorption of two photons will be enough to activate the disproportion reaction leading to [Ma + H]^+^ and [Ma − H]^−^, that is, the reactants for the analyte ionization.

## Figures and Tables

**Table 1:** Dissociation energy (*E*) of aromatic carboxylic acid dimers (see Scheme 2).

Dimers	*E* (eV)	*E*_337_ (eV)	*E*_355_ (eV)
Benzoic acid	7.03	3.68	3.50
2,5-dihydroxybenzoic acid	6.71		
4-methoxybenzoic acid	6.79		
4-nitrobenzoic acid	6.94		
4-N,N-dimethylaminobenzoic acid	6.56		

**Table 2:** Dissociation energy for the process described in Scheme 3.

Dimers	*E* (eV)	*E*_337_ (eV)	*E*_355_ (eV)
Benzoic acid	3.69	3.68	3.50
2,5-dihydroxybenzoic acid	3.55		
4-methoxybenzoic acid	3.43		
4-nitrobenzoic acid	3.35		
4-N,N-dimethylaminobenzoic acid	3.42		

**Table 3:** Dissociation energy for the process described in Scheme 4.

Dimers	*E* (eV)	*E*_337_ (eV)	*E*_355_ (eV)
Benzoic acid	1.06	3.68	3.50
2,5-dihydroxybenzoic acid	0.25		
4-methoxybenzoic acid	0.74		
4-nitrobenzoic acid	0.72		
4-N,N-dimethylaminobenzoic acid	0.77		

**Table 4:** Dissociation energy according to the model shown in Scheme 5.

Conjugate of TFA with	*E* (eV)	*E*_337_ (eV)	*E*_355_ (eV)
Benzoic acid	3.40	3.68	3.50
2,5-dihydroxybenzoic acid	3.86		
4-methoxybenzoic acid	3.90		
4-nitrobenzoic acid	3.26		
4-N,N-dimethylaminobenzoic acid	4.07		

**Table 5:** Dissociation energy of conjugates into a cation and TFA anion (see Scheme 5).

Conjugate of TFA with	*E* (eV)	*E*_337_ (eV)	*E*_355_ (eV)
Benzoic acid	6.04	3.68	3.50
2,5-dihydroxybenzoic acid	6.39		
4-methoxybenzoic acid	5.88		
4-nitrobenzoic acid	6.64		
4-N,N-dimethylaminobenzoic acid	5.48		

**Table 6:** Dissociation energy of conjugates into monomers.

Conjugate of TFA with	*E* (eV)	*E*_337_ (eV)	*E*_355_ (eV)
Benzoic acid	0.74	3.68	3.50
2,5-dihydroxybenzoic acid	0.70		
4-methoxybenzoic acid	0.76		
4-nitrobenzoic acid	0.73		
4-N,N-dimethylaminobenzoic acid	0.79		
